# Data-Informed and Accessibility-Oriented Motion Graphics for Depression-Related Health Communication in Aging Populations †

**DOI:** 10.3390/healthcare14121785

**Published:** 2026-06-20

**Authors:** Cong Mo, Khachakrit Liamthaisong, Jantima Polpinij

**Affiliations:** 1 Department of New Media, Faculty of Informatics, Mahasarakham University, Kantharawichai 44150, Thailand; 65011261006@msu.ac.th (C.M.); khachakrit.l@msu.ac.th (K.L.); 2 Department of Computer Science, Faculty of Informatics, Mahasarakham University, Kantharawichai 44150, Thailand

**Keywords:** short-form motion graphics, human–computer interaction, inclusive design, older adults, cognitive load, depression-related health communication

## Abstract

**Highlights:**

**What are the main findings?**

- The developed human-centered motion graphics system demonstrated relatively high usability with moderate cognitive load among older adults in depression-related health communication contexts.
- Visual clarity, structured content organization, and appropriate motion pacing were consistently identified as important factors for supporting accessibility and interaction quality among older adults.

**What are the implications of the main findings?**

- Human-centered and accessibility-oriented motion graphics may support more understandable and cognitively appropriate depression-related health communication for older adults.
- Integrating data-informed analysis with multi-stage evaluation may provide a practical framework for developing inclusive digital health communication systems that consider usability, accessibility, and user diversity.

**Abstract:**

**Background/Objectives**: Short-form motion graphics are increasingly used in digital health communication. However, limited research has examined their accessibility, interaction quality, and usability for older adults. This study explores the design and evaluation of short-form motion graphics as human–computer interaction systems for depression-related health communication in aging populations. **Methods**: A data-informed and human-centered approach was adopted, integrating clustering-based analysis, expert evaluation, and user-based assessment. Short-form motion graphics videos and user-generated comments were analyzed to identify design-related themes associated with accessible digital health communication. These insights informed the development of motion graphics prototypes. The evaluation involved independent expert groups and 200 older adult participants. Cognitive load and usability were assessed using a structured questionnaire. **Results**: The clustering analysis showed moderate cluster separation and provided an exploratory source of design insights. Expert evaluation highlighted the importance of visual clarity, structured content organization, and appropriate motion pacing. User evaluation yielded a mean usability score of 3.95 and a mean cognitive load score of 3.72, indicating generally positive perceptions of the developed motion graphics among participants. **Conclusions**: The findings suggest that combining data-informed analysis, expert review, and user evaluation may be useful for designing and assessing digital health communication systems for older adults. As this study was exploratory and did not include a control group, the findings should be interpreted within the context of the study and should not be considered evidence of causal relationships.

## 1. Introduction

Science and health communication play important roles in connecting scientific and health-related knowledge with the public by transforming complex ideas, findings, and evidence into forms that are understandable and accessible to non-specialist audiences [1,2]. Beyond the delivery of information, effective communication contributes to public understanding, informed decision-making, health literacy, and trust in scientific and healthcare information, particularly in situations involving uncertainty and social relevance [3,4]. As science, health, and society become increasingly interconnected, communication has evolved into a transdisciplinary field that integrates communication theory, science studies, digital health communication, and technology-mediated interaction [5,6,7,8]. From this perspective, communication can be viewed not only as media delivery but also as a system that supports users in accessing, processing, and understanding information.

The rapid growth of digital and networked media has transformed communication practices. Traditional one-way communication is increasingly complemented by interactive and participatory environments that support user-centered interaction, personalization, and flexible access to information [9,10,11]. These developments are closely related to human–computer interaction (HCI), where usability, accessibility, and interaction design influence how users engage with digital systems. Among various digital media formats, short-form content has become particularly prominent in mobile and social media environments, shaping how information is produced, distributed, and consumed [12,13,14,15,16]. This trend is also evident in digital health communication, where information must often be delivered efficiently to diverse user groups.

Short videos and visual communication formats are widely used in science and health communication because they can present information in a concise and engaging manner [17,18,19]. Previous studies have shown that these formats can attract attention, improve message retention, and support interaction more effectively than text-based communication alone [20,21,22,23]. Visual representations, animation, and multimodal elements may also facilitate information processing by organizing content and reducing unnecessary cognitive demands [24,25]. These characteristics are particularly relevant when communicating with users who have different cognitive and perceptual abilities. However, many existing studies have focused on general audiences or student populations. Comparatively fewer studies have examined how older adults interact with short-form digital health communication, despite well-documented age-related differences in attention, perception, and information processing [26,27].

As populations age worldwide, effective health communication becomes increasingly important [1,3,27]. Older adults may experience changes in attention, processing speed, and visual perception, which can influence how information is accessed, interpreted, and retained in digital environments [26,27]. These challenges may become more pronounced when communication topics require both cognitive engagement and emotional sensitivity [4,24]. Therefore, communication systems designed for aging populations should consider accessibility, clarity, and usability as essential design requirements [15,26,27].

Among various health communication topics, mental health presents additional communication challenges because information is often associated with stigma, emotional sensitivity, and limited public awareness [3,4]. These challenges may be particularly relevant for older adults, who may be less likely to seek information or openly discuss mental health concerns [27]. As a result, communication approaches that emphasize clarity and accessibility warrant further investigation in the context of mental health communication for older adults [15,26,27].

This study focuses on depression-related communication for aging populations. Depression is one of the most common mental health conditions among older adults and is often associated with reduced quality of life, social isolation, and delayed help-seeking. At the same time, symptoms may be under-recognized, and communication barriers may limit awareness and access to relevant information. These challenges highlight the need for communication formats that are both accessible and cognitively appropriate. In this context, short-form motion graphics are explored as a potential medium for presenting depression-related information in a structured and understandable manner.

Motion graphics represent one format that can be used for mental health communication because they combine visual, textual, and auditory elements within a structured presentation format. These characteristics allow information to be presented through multiple channels within a structured format. Such features may be relevant when presenting sensitive topics such as depression to older adults with diverse cognitive and perceptual characteristics.

Motion graphics have become a widely adopted approach for presenting information in digital environments. They involve the dynamic presentation of visual elements, including text, icons, illustrations, and infographics, arranged over time to support structured information delivery [14,28,29]. In health communication, health education, and public awareness campaigns, motion graphics can help organize information and guide user attention. Previous studies have suggested that animated visual cues may influence how users attend to and interact with information in digital environments [30,31,32,33]. These characteristics are particularly relevant for older adults and other users with diverse cognitive and perceptual needs.

Despite their growing use, several limitations remain in the literature. First, many studies focus on platform-level factors such as dissemination strategies, audience engagement, and visibility metrics rather than examining how communication systems are designed and experienced by users [34,35,36,37]. Second, the diversity of user characteristics, particularly among older adults, is often insufficiently addressed despite documented differences in cognitive and perceptual capabilities [26,27]. Third, motion graphics are frequently treated as creative or stylistic outputs rather than as technology-enabled communication systems that can be systematically designed and evaluated within a human-centered framework.

These limitations highlight the need to incorporate principles from universal design and human–computer interaction into digital health communication systems. Universal design emphasizes the development of systems that accommodate diverse user characteristics, including differences in cognition, perception, digital literacy, and interaction preferences [38]. From an HCI perspective, communication systems can be examined in relation to accessibility, usability, and interaction quality. In this study, universal design principles are reflected through readable typography, clear visual contrast, structured content segmentation, and multimodal presentation. Cognitive load theory is used as a conceptual lens for considering how information is organized and presented within the proposed system. Together, these principles informed the design and evaluation of the proposed motion graphics system.

Taken together, previous studies indicate growing interest in the use of short-form media for health communication. However, limited attention has been given to how motion graphics can be systematically designed and evaluated as human-centered communication systems for older adults. In particular, relatively few studies have examined the integration of data-informed design, usability, cognitive load, and accessibility considerations within a single evaluation framework. Addressing this gap requires moving beyond media-centered perspectives toward approaches that examine how information is structured, processed, and experienced within digital communication systems.

To address this gap, this study explores motion graphics as a technology-enhanced communication system designed with consideration of cognitive load and usability for aging populations. The proposed approach incorporates data-informed content preparation, including web-based data collection and clustering analysis, to identify communication-related themes that are subsequently considered during the design process. These analytical processes are not intended as standalone artificial intelligence systems but are used to support a human-centered design process. The contribution of this study lies not in developing new algorithms, but in examining how data-informed insights can be incorporated into the design and evaluation of digital health communication systems.

The study investigates how such a system relates to cognitive load and usability, which serve as indicators of interaction quality and information processing. Given the exploratory nature of this work, the focus is on understanding design characteristics and user perceptions rather than testing causal relationships or intervention effectiveness. To guide the investigation, the following research questions are proposed:

RQ1. What design-related themes can be identified from user-generated comments on short-form motion graphics through clustering analysis?

RQ2. How can the identified themes be translated into human-centered motion graphics design specifications for depression-related communication among older adults?

RQ3. How do older adults perceive the usability and cognitive load of the developed motion graphics?

The objective of this study is to examine motion graphics-based communication from a system-oriented perspective, focusing on how design principles derived from cognitive theory and user-centered considerations can be applied in the design and evaluation of digital health communication systems. The study does not seek to evaluate instructional effectiveness or promote a specific media format. Instead, it examines how communication systems can be designed to align with user diversity, accessibility needs, and interaction requirements in digital health communication contexts.

This study makes three contributions. First, it examines motion graphics from the perspectives of human–computer interaction, accessibility, and universal design. Second, it reports user perceptions of usability and cognitive load in the context of depression-related communication for older adults. Third, it describes a design and evaluation process that combines data-informed analysis, expert review, and user-based assessment. These contributions are specific to the context examined in this study.

The remainder of this paper is organized as follows. Section 2 reviews the related work. Section 3 presents the research methodology. Section 4 reports the evaluation results and discussion, including the limitations of the study. Finally, Section 5 concludes the paper.

## 2. Related Work

### 2.1. Science Communication in Digital and Interactive Media Systems

Science and health communication in digital environments can be understood not only as media processes but also as technology-mediated interactions between users and information systems [1,3,5]. In such environments, communication is increasingly shaped by user interaction, feedback, and participation across digital platforms [9,10,11,12].

Interactive and networked media environments enable dynamic exchanges between content and users, transforming communication into a more user-centered process rather than a one-way dissemination model [13,14]. This shift highlights the importance of understanding how users interact with information, particularly in short-form digital media where cognitive processing, usability, and accessibility become important considerations [15,16].

Short-form communication formats are widely used in science and health-related communication because they allow information to be delivered in concise and visually engaging ways [17,18,19,20,21,22,23]. Prior studies have examined dissemination efficiency, audience engagement, and communication reach in short-form media environments. However, comparatively less attention has been given to interaction mechanisms and system-level design factors that influence how users perceive, process, and understand information.

In short-form digital environments, user interaction is often influenced by limited attention span and rapid information consumption patterns [15,16]. These conditions create additional challenges for designing communication systems that are cognitively efficient, accessible, and easy to interact with, particularly for users with diverse cognitive and perceptual characteristics. While existing studies frequently emphasize dissemination and engagement metrics, fewer studies have examined how interaction structure and system-level design influence user experience and information processing in digital communication environments.

### 2.2. Motion Graphics as Human–Computer Interaction Interfaces

Motion graphics can be interpreted as human–computer interaction (HCI) interfaces that mediate communication between users and digital systems through structured visual representations [14,28,29]. Rather than functioning solely as aesthetic elements, motion graphics may also operate as interactive components that guide user attention and support information processing.

Previous studies have shown that animation and visual cues can influence user perception, attention allocation, and comprehension in digital environments [24,39,40,41]. These findings suggest that motion graphics can be understood as part of an interaction design process, where temporal sequencing and visual encoding contribute to usability, accessibility, and cognitive efficiency.

However, existing research often treats motion graphics primarily as stylistic or engagement-oriented media rather than as interaction systems that can be evaluated using user-centered criteria such as usability and cognitive load. As a result, comparatively less attention has been given to how motion graphics support interaction quality and information processing across diverse user groups.

From a system-oriented perspective, motion graphics may be viewed as interaction mechanisms that influence how users access, interpret, and respond to information in digital environments. This perspective is particularly relevant in contexts where user characteristics, such as cognitive capacity, perceptual ability, and age-related differences, may affect interaction quality. Despite this relevance, limited research has examined motion graphics as human-centered interaction systems that explicitly consider user diversity and accessibility in digital communication contexts.

### 2.3. Cognitive Load, Usability, and User Diversity in Communication Systems

Cognitive load theory provides a theoretical foundation for understanding how users process information under conditions of limited cognitive capacity [33,42,43,44]. In digital communication systems, effective design should minimize unnecessary cognitive load while supporting meaningful information processing [30,31].

Usability is closely related to cognitive load because it reflects how easily users can interact with and understand a system [43]. Higher usability is generally associated with reduced cognitive effort and more efficient interaction [45].

In aging populations, cognitive and perceptual diversity becomes an important consideration. Older adults may experience differences in attention, visual perception, and processing speed [46,47], which can influence how they interact with digital systems. These variations highlight the importance of design approaches that accommodate diverse user characteristics and support accessibility across different user groups. Such considerations are consistent with principles of universal design and inclusive human-centered systems [41,48].

Despite the importance of these factors, limited research has systematically examined how cognitive load and usability can be jointly applied in evaluating digital communication systems for diverse user groups, particularly aging populations [49,50,51].

From a human-centered system perspective, cognitive load and usability can serve as practical indicators of interaction quality and information processing. Designing for diverse user characteristics, especially in aging populations, requires approaches that align communication design with users’ cognitive and perceptual capabilities. In this regard, integrating cognitive load and usability may provide a useful basis for evaluating accessible and inclusive communication systems in digital environments.

### 2.4. Research Gaps and Study Positioning

In summary, prior research has established the importance of digital media in science and health communication, the role of motion graphics in visual representation, and the relevance of cognitive load and usability as evaluation criteria [3,8,28,29,43]. Existing studies have also demonstrated the potential of short-form digital media to support user engagement and information delivery [16,19,34,35]. However, much of the literature primarily focuses on dissemination outcomes, engagement metrics, or visual presentation aspects [16,19,20,21,22,23].

A closer review of previous studies reveals two important gaps. First, many studies adopt an engagement-oriented perspective that emphasizes metrics such as views, likes, and interaction frequency [16,19,34,35]. In contrast, relatively fewer studies examine communication systems from a system-oriented perspective, including how information is structured, processed, and interacted with from usability and cognitive perspectives [13,14,15]. As a result, interaction quality and information processing mechanisms remain comparatively underexplored [14,15,43].

Second, existing research predominantly focuses on general or student populations, while limited attention has been given to aging populations despite well-documented differences in cognitive processing, attention, and perceptual characteristics [27,46,47]. Consequently, design principles derived from general user groups may not fully reflect the interaction needs of older adults in digital communication environments [14,27,49,50].

These limitations highlight the need for human-centered and inclusive approaches that consider both interaction quality and user diversity [38,41,48]. In particular, there remains limited system-level research that conceptualizes motion graphics as interaction systems that can be evaluated using cognitive load, usability, accessibility, and user-centered design principles [28,29,43,44,48].

From this perspective, communication systems for aging populations should not only support information delivery but also accommodate differences in cognitive and perceptual capabilities [46,47,49,50]. This consideration is especially important in digital health communication contexts, where clarity, accessibility, and interaction quality may influence how users interpret and engage with sensitive information [3,17,19,27,49].

To address these gaps, this study examines motion graphics as a technology-enhanced communication system designed from a human-centered perspective. The study focuses on cognitive load and usability as indicators of interaction quality and explores how system-oriented design approaches may support accessible and inclusive communication for aging populations [24,38,41,43,48].

## 3. The Research Method

This section describes the methodological framework adopted in this study, including the processes of data collection, design development, and evaluation. The study combines data-informed analysis with human-centered design principles to develop and evaluate short-form motion graphics as digital communication systems for aging populations.

The methodology consists of three main stages. The first stage involves the extraction of design-related insights from user-generated data. The second stage focuses on the development of short-form motion graphics based on these insights. The third stage involves evaluation and refinement through expert review and user-based assessment. These stages were designed to support the examination of usability, cognitive load, accessibility, and interaction quality within the proposed communication system.

### 3.1. Method Overview

The methodological framework of this study follows a sequential process that integrates data-informed design and human-centered evaluation. The overall workflow consists of three main phases.

In the first phase, user-generated data were collected from short-form motion graphics videos on YouTube and analyzed using clustering techniques to identify design-related patterns. This process was intended to provide initial design insights based on real user feedback and interaction-related responses.

In the second phase, the extracted insights were translated into design specifications and applied to the development of short-form motion graphics videos. The design process was guided by principles related to health communication, human–computer interaction, cognitive load, and usability.

In the third phase, evaluation was incorporated into the design process. Expert-based evaluation was conducted to assess design appropriateness and support content refinement. This stage was followed by user-based evaluation involving older adult participants to examine cognitive load and usability. Participant selection and measurement instruments are described in Section 3.4.

This framework was designed to support a structured investigation of how data-informed design relates to interaction quality in short-form digital communication systems for aging populations. The study does not include a control group because the primary objective is to examine interaction quality and usability from a system-oriented perspective rather than to compare intervention effectiveness. Accordingly, the methodology follows an exploratory and design-oriented approach aimed at examining interaction patterns and user-centered communication characteristics rather than establishing causal relationships.

The overall study design consists of three sequential stages: (1) extraction of design insights from user-generated data through clustering analysis, (2) development of motion graphics based on these insights, and (3) multi-stage evaluation involving independent expert groups and a user group (*n* = 200).

### 3.2. Data Collection and Initial Design Insights: Clustering Analysis

To obtain data-informed design insights, short-form motion graphics videos were collected from YouTube. The selection criteria included: (1) relevance to science or health communication, (2) the use of motion graphics as the primary visual presentation approach, and (3) a minimum threshold of 10,000 likes. The threshold was not used as an indicator of design quality. Rather, it was applied as a practical sampling criterion to identify videos that had generated substantial user interaction and a sufficient volume of public comments. Because the clustering analysis relied on user-generated textual feedback, videos with larger numbers of interactions were more likely to provide an adequate amount of comment data for exploratory analysis. The threshold was therefore used to support data collection rather than to evaluate communication effectiveness or content quality.

A total of 20 videos meeting these criteria were selected. The videos demonstrated moderate to high levels of audience engagement in terms of views, likes, and comments [43]. These characteristics suggest that the dataset was suitable for exploratory analysis of user feedback patterns related to communication and interaction. The number of selected videos was considered appropriate because the objective of this study was to identify interpretable design-related patterns rather than achieve statistical representativeness. In addition, the videos varied in content style, presentation format, and audience interaction characteristics, allowing a broader range of user responses to be examined.

The video selection process was conducted between January and March 2025 using the YouTube search function and recommendation system. Search terms related to health communication, depression awareness, mental health education, and motion graphics were used. Examples included “depression awareness animation”, “mental health motion graphics”, “health communication animation”, and similar keyword combinations. Only publicly accessible videos were considered.

To improve consistency, the analysis focused on videos presented in English or Chinese because these languages could be reliably reviewed during the data collection process. Videos that primarily consisted of live-action recordings, interviews, or slide presentations without substantial motion graphics elements were excluded.

A final set of 20 videos meeting the predefined selection criteria was retained for analysis. The selected videos represented different presentation styles and communication approaches within the scope of science and health communication.

User comments associated with the selected videos were collected using the YouTube Data API [44]. In cases where API access was limited because of quota restrictions or data availability, supplementary manual collection was conducted. Approximately 2000 comments were obtained and used as the primary dataset for analysis. Comments were collected from all selected videos using the available YouTube Data API retrieval process. When videos contained a very large number of comments, the accessible comments returned through the collection process were used. No sampling based on sentiment, popularity, or user characteristics was performed. After collection, duplicate entries, non-substantive comments, spam-like content, and incomplete records were removed during data preprocessing. The resulting dataset contained approximately 2000 comments that were retained for clustering analysis.

After preprocessing and removal of duplicate, incomplete, and non-informative entries, the final dataset consisted of approximately 2000 comments collected from 20 videos. The comments varied in length and content, including observations related to visual presentation, animation characteristics, communication style, emotional responses, and general audience reactions.

To identify design-related themes, the textual data were analyzed using a clustering-based approach. The purpose of this analysis was not only to summarize user feedback but also to explore interpretable patterns that could support communication design and interaction-oriented development.

The preprocessing pipeline followed standard natural language processing procedures. First, all comments were converted to lowercase to maintain consistency across the dataset. Tokenization was then applied to segment the text into individual terms. Stop words were removed to reduce the influence of high-frequency but semantically weak terms, and stemming was performed to reduce words to their root forms. Non-textual elements, including emojis and URLs, were removed or normalized where appropriate.

Following preprocessing, two feature representation approaches were used to capture different characteristics of textual semantics. First, TF–IDF (Term Frequency–Inverse Document Frequency) was applied to represent the relative importance of terms within the corpus. This representation provides an interpretable and sparse feature structure that highlights discriminative keywords across documents. Second, Word2Vec-based embeddings were generated to capture semantic relationships among words within a dense vector space. Using both approaches allowed the analysis to consider both frequency-based and semantic-based representations of user comments.

Clustering analysis was conducted using three algorithms: (1) K–means clustering [52], (2) Agglomerative hierarchical clustering [52], and (3) DBSCAN (Density-Based Spatial Clustering of Applications with Noise) [52]. K–means was selected because of its efficiency and relatively interpretable cluster structures. Agglomerative clustering was included to examine hierarchical relationships among data points, while DBSCAN was used to identify potential noise and irregular patterns within the dataset.

For K–means clustering, the number of clusters (k) was evaluated within the range of *k* = 2 to 6. The selection of k was guided by both the elbow method and average silhouette analysis. The elbow method was used to examine changes in within-cluster variation across different values of *k*, while average silhouette scores were calculated to assess cluster cohesion and separation. Both analyses suggested that *k* = 3 provided a reasonable balance between cluster compactness and interpretability for the present dataset. Consequently, *k* = 3 was selected for subsequent analysis.

Clustering performance was assessed using internal validation metrics, including the Silhouette Score (S.S.) [53] and the Davies–Bouldin Index (DBI) [53]. The results indicated that a three-cluster solution (*k* = 3) provided a suitable balance between thematic differentiation and interpretability. Similar clustering structures were observed across multiple runs with different random initializations, supporting the consistency of the selected solution.

Comparisons across clustering methods showed that K–means produced clearer and more interpretable cluster structures for design-oriented analysis. Agglomerative clustering generated similar groupings but required greater computational complexity, whereas DBSCAN identified noise points but produced less stable cluster boundaries. Accordingly, K–means was selected as the primary method for subsequent analysis because the resulting clusters were easier to interpret in relation to communication design patterns. This decision is consistent with previous exploratory studies suggesting that moderate cluster sizes can support thematic differentiation while maintaining interpretability [45].

The clustering process was implemented using Python (version 3.9) with standard machine learning libraries, including scikit-learn. To improve stability, K-means clustering was performed with 20 random initializations (random_state = 42), and the solution associated with the most suitable internal evaluation metrics was selected.

The resulting clusters were interpreted using an iterative open-coding approach. Representative keywords and sample comments from each cluster were examined to generate initial thematic codes. These codes were refined through multiple iterations and subsequently grouped into higher-level design-related categories.

To examine the interpretability and practical relevance of the identified themes, an expert review process was conducted. Three domain experts (*n* = 3) with experience in motion graphics design, digital media, and communication systems participated in the review process. The experts were selected based on at least five years of professional experience in related areas to ensure domain-relevant expertise in evaluating communication and design patterns.

The expert review was conducted to provide an additional qualitative perspective on the interpretability of the identified themes. The role of the experts was not to establish statistical reliability but to examine whether the clustering outputs could be meaningfully interpreted within the context of motion graphics and communication design. Differences in interpretation were discussed collectively and documented during the review process. Accordingly, the expert review should be regarded as a supplementary interpretive procedure rather than a formal reliability assessment.

Three primary thematic categories were identified: (1) design-related feedback, (2) sentiment-related responses, and (3) general or unrelated comments. Among these categories, the design-related cluster served as the primary source of initial design insights for the subsequent development stage.

To provide additional context regarding the identified clusters, representative keywords associated with each cluster are presented in Table 1. These keywords illustrate the general thematic characteristics of the clusters and are intended to support interpretation of the clustering outputs.

The keyword distributions provide a descriptive overview of the thematic content associated with each cluster. These representations are intended to support interpretation of the clustering results and should not be considered exhaustive descriptions of cluster content.

Overall, the clustering-based analysis provided a data-informed foundation for communication design by supporting the translation of user feedback into practical design considerations while maintaining alignment with human-centered communication principles and accessibility-oriented design perspectives.

### 3.3. Short-Form Motion Graphics as Human–Computer Interaction Design

Based on the design insights obtained from the clustering analysis, short-form motion graphics videos were developed using a human-centered and system-oriented design approach. In this study, motion graphics are considered not only as visual media but also as human–computer interaction (HCI) interfaces that support communication between users and information systems through structured visual and temporal presentation.

The design process consisted of four main stages: (1) interpretation of clustering results, (2) extraction of design-related insights, (3) translation of insights into design specifications, and (4) development of short-form motion graphics prototypes.

In the first stage, the clustering results described in Section 3.2 were examined to identify recurring patterns within user-generated comments. Three primary clusters were identified: (1) design-related feedback, (2) sentiment-related responses, and (3) general comments. Among these, the design-related cluster served as the primary source of initial design insights. Representative keywords and comment excerpts within this cluster were reviewed through an expert-based interpretation process to identify recurring themes relevant to motion graphics design.

In the second stage, three domain experts with experience in motion graphics design and digital communication systems reviewed the clustered data to identify design-related patterns. The analysis focused on visual presentation, audio characteristics, content organization, and interaction-related feedback. Several recurring design considerations were identified from the design-related cluster. These considerations were derived through an interpretive review of representative comments and subsequently translated into practical design specifications. These included: (1) the use of high-contrast color schemes to support visual clarity, particularly for older adults; (2) moderate animation speed to support information processing without creating excessive cognitive demand; (3) clear and legible typography to improve readability; (4) synchronized audio narration to reinforce visual information; and (5) structured content sequencing to guide attention and support information processing. These observations are generally consistent with previous studies describing the role of visual cues, animation, and multimodal presentation in supporting attention and comprehension [24,30,31].

To improve transparency in the design process, representative comments from the design-related cluster were mapped to the corresponding design considerations identified during expert interpretation. These considerations were subsequently translated into design specifications used in the development of the motion graphics prototypes. Examples of this mapping process are presented in Table 2.

In the third stage, the identified insights were translated into design specifications guided by human-centered and universal design principles [38,41,48]. The design process was informed by three main considerations: (1) communication in interactive digital media environments, emphasizing structured, accessible, and user-oriented information delivery [3,10,17]; (2) motion graphics as human–computer interaction (HCI) interfaces that mediate interaction between users and visual information through dynamic visual representations and multimodal elements [14,28,29]; and (3) cognitive load, usability, and user diversity, particularly in relation to age-related differences in cognitive processing, sensory perception, and perceptual characteristics [26,27,33,46,47]. These considerations informed design decisions related to layout organization, visual hierarchy, animation pacing, and multimodal integration to support effective information processing and accessible user interaction [24,31].

In the final stage, short-form motion graphics videos were developed based on the defined specifications. The content focused on depression-related information for older adults, including symptom awareness and introductory guidance related to mental health conditions. Each video was designed with a duration suitable for short-form digital media contexts, generally under one minute, to align with common information consumption patterns in digital environments [16,19,34,35,36,37].

The content was organized into sequential segments, with each segment focusing on a single key message to support gradual information processing. Visual elements were arranged to guide user attention through temporal sequencing while minimizing unnecessary visual complexity. Audio narration was incorporated to complement visual information and support dual-channel information processing [24,32].

From an HCI perspective, the developed motion graphics can be viewed as interaction systems that influence how users access, interpret, and respond to information [14,28,29]. The design process emphasized clarity, consistency, accessibility, and cognitive efficiency rather than aesthetic complexity alone, reflecting established principles of usability and user-centered interaction design [15,43]. This approach was intended to support effective interaction across diverse user groups. Consideration of age-related differences in perception and cognition further reflects alignment with universal design and inclusive design principles intended to support broader accessibility in digital communication environments [38,41,46,47,48].

After the development stage, a dual evaluation approach was conducted consisting of expert-based evaluation and user-based evaluation. This approach was intended to assess design appropriateness and interaction quality from both design and user perspective.

(1) Expert-Based Evaluation

To examine the appropriateness of the developed short-form motion graphics, an expert-based evaluation was conducted prior to user testing. Three domain experts with experience in motion graphics design and digital communication systems participated in the evaluation process.

The evaluation focused on four dimensions: (1) visual design, including clarity, color contrast, and readability; (2) motion and timing, including animation speed and transition smoothness; (3) content organization, including logical flow and segmentation; and (4) interaction and usability, including ease of understanding and attention guidance.

Each dimension was assessed using a five-point Likert scale together with qualitative feedback. The purpose of this evaluation was to identify potential design issues and support refinement of the videos in alignment with human-centered design principles rather than to establish definitive performance claims. Feedback from the experts was subsequently used to revise and refine the videos before user-based evaluation [48].

(2) User-Based Evaluation

Following the expert evaluation, user-based evaluation was conducted with older adult participants. The evaluation focused on cognitive load and usability as indicators of interaction quality and user experience [43,49].

Cognitive load reflects the mental effort required for information processing, whereas usability reflects how easily users can interact with and understand the communication system. Together, these measures provide insight into how the developed motion graphics relate to information processing and interaction experience among aging populations.

Participants were asked to watch the short-form motion graphics videos and complete a structured questionnaire consisting of 12 items. Responses were recorded using a five-point Likert scale [54]. The collected data were analyzed using descriptive statistics, and internal consistency reliability was examined using Cronbach’s alpha.

**Figure 1:**
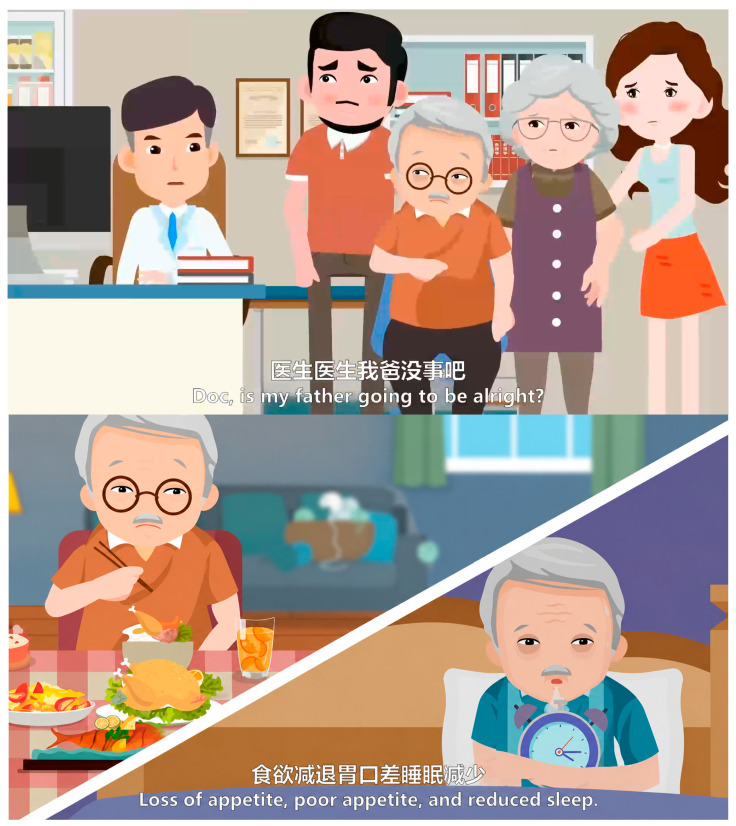
Example of structured visual presentation in the developed short-form motion graphics for depression-related communication among older adults.

Figure 1 illustrates examples of structured visual communication used in the developed short-form motion graphics for depression-related communication among older adults. The upper scene presents a healthcare communication context involving older adults and family members interacting with a physician, reflecting the emotional and informational aspects of depression-related consultation. The lower scene demonstrates symptom-focused visual presentation, including appetite reduction and sleep-related problems, using simplified illustrations, segmented layouts, and visual emphasis to support readability and information processing for aging populations. These examples reflect the application of human-centered and accessibility-oriented design principles in the proposed communication system.

### 3.4. Sampling, Participants, and Measurement Instrument

Participants were recruited using a purposive sampling approach targeting older adults aged 50 years and above in Nanning, China. This age threshold was selected to reflect the transition into aging populations in which changes in cognitive processing, attention, and perception may begin to influence interaction with digital systems [27,46,47].

A total of 200 participants were recruited and divided into two groups according to educational background: 100 participants with education above the secondary level and 100 participants below the secondary level. This grouping was included to allow additional examination of participant diversity within the study sample. Educational background was considered a potentially relevant characteristic because it may be associated with differences in experiences and interactions with digital information. By balancing the sample across educational levels, the study aimed to include participants with varied backgrounds within the target population.

To provide additional context regarding the study sample, demographic characteristics of the participants are summarized in Table 3. The information includes age, gender distribution, and educational background. These characteristics are reported to support interpretation of the user-based evaluation results and to provide a clearer description of the participant group involved in the study.

Additional participant characteristics are presented in Table 3. The participants represented a range of ages, educational backgrounds, and levels of digital media experience. Information regarding age, gender, and digital media use is provided to offer additional context for interpreting the user-based evaluation results.

Although the sampling approach was purposive and context-specific, it was considered appropriate for exploratory evaluation of interaction quality in aging populations. The objective of the sampling strategy was not to achieve statistical representativeness but rather to capture variability among users with different educational backgrounds and interaction characteristics. This approach is consistent with the exploratory and design-oriented nature of the study.

All participants provided informed consent prior to participation, and the study was conducted in accordance with ethical guidelines for human-subject research.

To evaluate the developed short-form motion graphics, a structured questionnaire consisting of 12 items was designed [54]. The questionnaire focused on two main constructs: cognitive load and usability. These constructs are commonly used as indicators of interaction quality in human–computer interaction research [43,45].

The decision to use 12 items was intended to balance measurement coverage and participant burden. Because the participants were older adults, the questionnaire was intentionally designed to remain concise in order to reduce fatigue, maintain attention, and support response consistency. Previous studies have suggested that shorter questionnaires may be more suitable for aging populations, particularly in perceptual and cognitive evaluation contexts [27,49].

The questionnaire consisted of two sections:

(1) Cognitive Load (6 items): assessing perceived mental effort, information complexity, and ease of understanding.
(2) Usability (6 items): assessing clarity of presentation, ease of interaction, navigation flow, and overall user experience.

All items were measured using a five-point Likert scale ranging from 1 (strongly disagree) to 5 (strongly agree). The wording of the questionnaire items was simplified to improve clarity and readability for older participants. Technical terminology and complex sentence structures were avoided where possible.

For the cognitive load items, higher scores indicate greater perceived mental effort and information processing demands. For the usability items, higher scores indicate more positive perceptions of usability and interaction quality. No reverse-coded items were included in the questionnaire. Scores for each construct were calculated by averaging the responses across the corresponding six items. The resulting mean scores were used for descriptive and comparative analyses.

The questionnaire design was informed by principles of cognitive load theory and usability evaluation. The instrument focused on perceived interaction quality rather than objective performance outcomes. Prior to deployment, the questionnaire was reviewed by domain experts to assess content relevance and clarity. The complete questionnaire is provided in Appendix A for transparency and reproducibility.

The collected data were analyzed using descriptive statistics, including mean and standard deviation, to summarize participant responses. Internal consistency reliability was examined using Cronbach’s alpha.

Given the exploratory nature of the study, the analysis focused on identifying general patterns and tendencies in user perception rather than testing predefined hypotheses. The study was designed as an early-stage evaluation of interaction quality and user experience rather than as a comparative intervention study. Descriptive statistics were used to summarize participant responses. In addition, independent-samples t-tests were conducted to provide supplementary information regarding potential differences between participants with different educational backgrounds. The findings were interpreted as indicative patterns within the study context and should not be considered evidence of causal relationships or statistically generalizable effects.

Different expert groups were involved across the design and evaluation stages to support independent assessment throughout the study process.

## 4. Evaluation and Discussion

This section presents the findings from the evaluation process, including clustering analysis, expert validation of the extracted design insights, expert evaluation of the developed videos, and user-based evaluation involving older adult participants. The results are presented using descriptive statistics and qualitative interpretation to examine interaction quality, usability, and user perception patterns within the proposed communication system. Given the exploratory and design-oriented nature of the study, the findings are interpreted as indicative patterns rather than a definitive conclusion.

### 4.1. Clustering Results and Validation

The clustering analysis was conducted to identify recurring patterns within user-generated comments associated with short-form motion graphics. Before comparing clustering algorithms, an initial analysis was performed to determine an appropriate number of clusters for K-means clustering. The objective was to identify a cluster structure that balanced thematic differentiation and interpretability while remaining suitable for exploratory analysis.

Table 4 presents the average silhouette scores obtained from K-means clustering using different values of k. Among the evaluated configurations, *k* = 3 achieved the highest silhouette score (0.42), indicating relatively better cluster cohesion and separation than the other values of *k*.

Based on this result, *k* = 3 was selected for subsequent analysis. This configuration provided the highest silhouette score and was therefore used in the clustering experiments reported in this study.

After selecting *k* = 3 for K-means clustering, additional experiments were conducted to compare clustering algorithms and feature representation methods. The objective was to examine whether different analytical configurations produced consistent and interpretable cluster structures.

Table 5 compares the clustering results obtained from K-means, Agglomerative clustering, and DBSCAN using TF–IDF and Word2Vec representations. For K-means and Agglomerative clustering, the number of clusters was defined as *k* = 3. DBSCAN was implemented using density-based parameters to examine alternative cluster structures within the dataset.

The clustering performance obtained from different algorithms and feature representations is presented in Table 5. Among the evaluated configurations, K-means with TF–IDF achieved the highest Silhouette Score (0.42) and the lowest Davies–Bouldin Index (1.68). These values should be interpreted comparatively across the evaluated configurations rather than as evidence of strong cluster separation. The remaining configurations produced lower silhouette scores and higher Davies–Bouldin Index values.

Agglomerative clustering produced results that were generally comparable to those of K-means, although the corresponding evaluation metrics were slightly lower. DBSCAN produced lower silhouette scores and higher Davies–Bouldin Index values than the other evaluated methods. These results provide a comparative view of clustering behavior across different algorithms and feature representations within the present dataset.

Across the evaluated feature representations, TF–IDF was associated with higher silhouette scores and lower Davies–Bouldin Index values than Word2Vec. Within the context of this study, TF–IDF provided the most favorable combination of internal evaluation metrics among the evaluated configurations.

Overall, the clustering results are consistent with the exploratory objective of identifying interpretable design-related patterns rather than establishing statistically distinct cluster boundaries. Although the cluster separation was moderate, the resulting groupings were used as the basis for subsequent thematic interpretation and design analysis.

To further examine the interpretability of the clustering outputs, the identified clusters were reviewed by three independent domain experts. The review focused on whether the resulting groupings could be meaningfully interpreted within the context of motion graphics and communication design. This review process was qualitative in nature and was conducted as a supplementary interpretive procedure rather than a formal reliability assessment.

Taken together, the clustering results and expert review provided the basis for selecting K-means with TF–IDF for the subsequent stages of analysis. This configuration was retained for further thematic interpretation and design development within the scope of the present study.

### 4.2. Validation of Extracted Design Insights

The design-related cluster was further analyzed to extract design-relevant features associated with accessible and human-centered motion graphics design. These extracted features were then evaluated by three independent experts to assess their relevance and reliability in the context of inclusive digital health communication. Table 6 summarizes the expert validation results.

As shown in Table 6, all extracted design features received mean scores above 4.0. These results indicate generally positive evaluations from the expert reviewers. Differences across features provide additional information regarding how the identified design characteristics were assessed within the context of this study.

Typography readability (Mean = 4.50) and color contrast clarity (Mean = 4.33) received the highest ratings among the evaluated design features. These results suggest that the experts assigned relatively high scores to visual presentation features. Content segmentation (Mean = 4.27) also received a comparatively high rating, while animation speed (Mean = 4.17) and audio synchronization (Mean = 4.10) received slightly lower scores.

Animation speed and audio synchronization also showed greater variability in expert ratings (SD ≈ 0.70–0.76) compared with the other features. This observation indicates that expert opinions regarding these elements were somewhat more varied within the evaluation process.

Overall, all evaluated design features received mean scores above 4.0. Differences in the reported scores are presented to describe the distribution of expert ratings across features. Because the expert evaluation involved a small number of reviewers and was not intended for statistical comparison, the results should not be interpreted as evidence that one feature was significantly more important than another.

The evaluated features were subsequently considered during the design development process. The expert ratings were used as one source of input alongside the clustering results and design review process. The ratings were not used to rank features according to statistical importance but rather to provide additional perspectives during design refinement.

While the results provide expert perspectives on the identified design features, they should be interpreted within the context of the present study. The findings are intended to support the design process and should not be considered prescriptive or universally applicable.

### 4.3. Expert Evaluation of Developed Motion Graphics

Following the design phase, the developed short-form motion graphics videos were evaluated by a separate group of three experts to assess their quality prior to user testing. The evaluation focused on four dimensions: visual design, motion and timing, content structure, and interaction and usability. The results are summarized in Table 7.

As shown in Table 7, all dimensions received mean scores above 4.0. These results indicate generally positive evaluations from the expert reviewers. At the same time, differences across dimensions provide additional context for understanding how the developed videos were assessed.

Content structure received the highest mean score (Mean = 4.32). This result indicates that the organization and sequencing of information were evaluated positively by the experts. Visual design (Mean = 4.28) and interaction and usability (Mean = 4.20) also received relatively high ratings, suggesting favorable evaluations of the presentation and overall design characteristics of the videos.

Motion and timing received the lowest mean score among the evaluated dimensions (Mean = 4.15) and showed the highest variability (SD = 0.71). Compared with the other dimensions, expert opinions regarding animation pacing were somewhat more varied. This observation may indicate differences in expert preferences or perspectives regarding temporal presentation.

Overall, the expert evaluation results show positive assessments across all evaluated dimensions. Content structure and visual design received the highest ratings, while motion and timing showed greater variation in expert responses. These results provide additional context for interpreting the subsequent user-based evaluation findings.

A separate expert group was involved at this stage of the study. This approach provided an independent review of the developed videos and allowed the evaluation process to be conducted separately from the earlier stages of design development.

### 4.4. User-Based Evaluation

The developed videos were evaluated by 200 participants using a 12-item questionnaire. Reliability analysis indicated acceptable internal consistency (Cronbach’s alpha = 0.84). Cronbach’s alpha was calculated across all 12 questionnaire items to assess the overall internal consistency of the instrument. The resulting value of 0.84 indicates acceptable reliability for the questionnaire used in this study.

Table 8 summarizes the overall results for cognitive load and usability. The evaluated videos presented depression-related information designed for older adults.

As shown in Table 8, the mean cognitive load score was 3.72 (SD = 0.81), while the mean usability score was 3.95 (SD = 0.74). These results provide an overview of participant perceptions of the developed motion graphics. The standard deviation values indicate some variation in responses across participants, particularly for cognitive load. 

The difference between the cognitive load and usability scores provides additional context for interpreting the evaluation results. Usability received a higher mean score than cognitive load. These results reflect the participant responses collected in the present study and provide an overall view of how the developed motion graphics were perceived.

Both mean scores were above the neutral midpoint of the five-point scale (3.0). Within the context of this exploratory study, these values suggest generally positive user perceptions of the developed motion graphics. However, the results should be interpreted descriptively because the study was not designed to establish benchmark performance levels or compare alternative communication formats.

Variation in cognitive load responses was observed across participants. In contrast, usability ratings were relatively more consistent within the study sample. In contrast, usability ratings were relatively more consistent within the study sample. These results indicate that participant responses varied across individuals within the study context.

To further examine potential differences between educational groups, independent-samples t-tests were conducted for both cognitive load and usability scores. Participants were categorized into two groups based on educational background: above secondary education and below secondary education. The results are presented in Table 9.

As shown in Table 9, no statistically significant differences were observed between participants with different educational backgrounds for either cognitive load or usability (*p* > 0.05). The mean scores were comparable across the two groups, indicating broadly similar response patterns within the study sample.

Several explanations may account for the absence of statistically significant differences between the educational groups. One possibility is that the design characteristics of the developed motion graphics were sufficiently clear and structured to support users with different educational backgrounds. It is also possible that educational background was less influential than other factors, such as prior digital media experience or individual differences in information processing. Because the study was exploratory and relied on a concise questionnaire, these findings should be interpreted cautiously and may warrant further investigation in future studies.

These results provide additional context for interpreting the descriptive findings. Although participants differed in educational background, the reported perceptions of cognitive load and usability were relatively similar. Given the exploratory nature of the study, these results should be interpreted as supplementary evidence within the study context rather than as evidence of causal relationships or statistically generalizable effects.

When considered together with the expert evaluation results, several design characteristics were identified across different stages of the study, including visual clarity, content organization, and presentation structure. These characteristics were also reflected in the design specifications applied during the development process.

Overall, the results provide an overview of participant perceptions of the developed motion graphics. Differences in cognitive load responses were observed across participants, indicating variation in how the information was experienced within the study sample. These observations highlight the importance of considering user diversity when designing digital health communication systems for aging populations.

### 4.5. Discussion

A data-informed and human-centered approach was used in the design and evaluation of short-form motion graphics for aging populations. By combining clustering analysis, expert review, and user-based evaluation, the study provides a context-specific description of how these methods were applied within the development process. The findings are interpreted within the scope of the present study and should not be generalized beyond the evaluated context.

The clustering analysis shows that user-generated data can be used to explore design-relevant patterns in a way that remains interpretable. Although the clusters are not sharply separated, they can be considered sufficiently coherent for supporting design-oriented interpretation. The inclusion of expert validation further suggests that these patterns are meaningful for guiding communication and accessibility-oriented design decisions, even in the absence of strong statistical separation. This points to the value of combining computational methods with human judgment, particularly in health communication contexts where interpretability, usability, and clarity of information presentation may be more important than model complexity.

The expert evaluation highlights several design elements that are consistently perceived as important, particularly visual clarity and structured content organization. These findings align with established principles in human–computer interaction, universal design, accessible health communication, and health literacy-oriented communication, where clarity, consistency, and readability support interaction across users with varying cognitive, perceptual, and literacy-related characteristics.

The user-based results further indicate that the developed motion graphics provide a generally usable interaction experience, while still requiring a moderate level of cognitive effort. This reflects the challenge of presenting health-related information in short-form formats, where clarity must be balanced with information density. Variations in cognitive load across participants also suggest that differences in background, familiarity with digital media, health information interpretation, and individual processing capacity influence how information is interpreted. This is particularly relevant in depression-related communication for older adults, where pacing, structure, and clarity may affect how sensitive health information is understood and accessed.

The observed pattern of relatively high usability together with moderate cognitive load is generally consistent with previous research on multimedia learning and animated communication. Prior studies have suggested that structured visual presentation, clear visual cues, and multimodal information delivery may support information processing while reducing unnecessary cognitive demands [24,30,31]. Studies involving older adults have also emphasized the importance of readability, visual clarity, and information organization in supporting interaction with digital health information [26,46,47]. Although direct comparisons are not possible because of differences in study designs and evaluation measures, the present findings are broadly consistent with these observations.

Alternative explanations should also be considered when interpreting these findings. Because the study did not include a comparison condition, it is not possible to determine whether the reported perceptions were specific to the developed motion graphics or would have been observed with other communication formats. In addition, participant responses were based on self-reported evaluations and may have been influenced by response tendencies or social desirability effects. These considerations should be considered when interpreting the results.

At a broader level, these findings relate to ongoing discussions in aging societies, where digital communication systems need to accommodate a wide range of user needs and accessibility considerations. Rather than relying on a single design approach, the results suggest the importance of considering cognitive load, usability, perceptual accessibility, communication clarity, and health information accessibility together as part of inclusive system design. This is especially important in health communication contexts such as depression, where understandable and accessible information may support more inclusive communication experiences and improve engagement with health-related information among aging populations.

The contribution of this study does not lie in proposing new algorithms or artificial intelligence models, but in demonstrating how data-driven analytical techniques can be integrated into human-centered design processes for digital health communication systems. In this role, computational methods help identify patterns in large datasets, which are then refined through expert input and evaluated through user interaction and accessibility-oriented assessment. This perspective may be relevant for the design and evaluation of other healthcare communication systems that involve diverse user populations.

A further strength of the study lies in the use of independent expert groups at different stages of evaluation. Separate groups were involved in validating clustering results, assessing extracted design insights, and evaluating the developed motion graphics. This approach helps reduce the risk of bias and allows each stage to be examined from a focused perspective, contributing to a more balanced evaluation process for human-centered communication design in healthcare-related contexts.

Taken together, the findings describe the outcomes observed within the present study. The combination of clustering analysis, expert review, and user-based evaluation provided multiple perspectives during the design and evaluation process. Because the study was exploratory and context-specific, the results should be interpreted as observations from the examined setting rather than as evidence of effectiveness for broader communication contexts.

From a practical design perspective, the findings suggest several considerations for developing motion graphics for older adults. Information may be easier to follow when content is presented in short segments, visual contrast is maintained between foreground and background elements, and animation pacing remains moderate rather than rapid. In addition, synchronized narration and clear typography may support readability and information processing. These observations are intended as design considerations derived from the present study rather than as prescriptive design standards.

### 4.6. Limitations

Several limitations should be considered when interpreting the findings of this study.

First, the clustering results showed only moderate separation between clusters. Although the resulting groupings were useful for thematic interpretation and design development, some overlap between clusters may exist. Therefore, the identified clusters should be interpreted as exploratory analytical groupings rather than as clearly distinct categories.

Second, the study relied on self-reported measures of cognitive load and usability. Participant responses may have been influenced by subjective perception, response tendencies, and individual differences. In addition, because the evaluation relied on self-reported responses, the possibility of social desirability bias cannot be excluded. Some participants may have been less willing to express negative opinions about health-related communication materials, which may have influenced the reported usability perceptions.

Variations in prior experience with digital media, health-related information, and communication formats may also have affected how participants interpreted and responded to the questionnaire.

Third, the participant sample was limited to older adults in a specific geographic and cultural context. While this focus was consistent with the objectives of the study, it may limit the transferability of the findings to other populations, settings, or health communication contexts. Differences in healthcare system familiarity, access to health information, and attitudes toward mental health and depression may vary across regions and cultural settings. These contextual factors were not explicitly examined in the present study and may influence how communication materials are perceived and interpreted. In addition, the study examined perceptions of interaction quality and usability rather than clinical understanding, knowledge retention, or mental health outcomes.

Fourth, the study did not include a comparison condition. The developed motion graphics were evaluated as a standalone communication format and were not compared with static infographics, text-based materials, or alternative video formats. As a result, the specific contribution of motion graphics as a communication format cannot be isolated from the broader effects of content presentation. Future studies may benefit from comparative designs that examine different communication formats under similar conditions.

Fifth, although separate expert groups were involved across the clustering review, design evaluation, and user evaluation stages, the overall study remained closely connected to the design process conducted by the research team. Efforts were made to support independent assessment through the use of different expert groups; however, the potential influence of researcher involvement cannot be completely excluded.

Finally, the study adopted a single design configuration and did not examine adaptive or personalized variations of motion graphics. Given the diversity of cognitive, perceptual, and literacy-related characteristics among older adults, future research may explore how alternative design configurations influence user experience and interaction quality across different user groups.

Taken together, these limitations indicate that the findings should be interpreted within the context of this exploratory and design-oriented study. The results provide insights into the design and evaluation process examined in this work but should not be interpreted as evidence of effectiveness beyond the specific context investigated.

## 5. Conclusions

This study explored the design and evaluation of short-form motion graphics for depression-related health communication among older adults. A data-informed design process was adopted, combining clustering analysis of user-generated comments, expert review, and user-based evaluation.

The clustering analysis identified several recurring themes related to visual presentation, content organization, animation pacing, audio support, and readability. These themes were used as inputs during the design process and were subsequently reflected in the developed motion graphics.

Expert evaluations provided generally positive assessments of the evaluated design features and video dimensions. User evaluation results indicated mean scores of 3.72 for cognitive load and 3.95 for usability. Additional analyses found no statistically significant differences between participants with different educational backgrounds.

Taken together, the findings provide a description of how the developed motion graphics were perceived within the context of this study. The results suggest that visual clarity, content organization, readability, and presentation structure may support understandable and relatively usable communication experiences for older adults. These considerations emerged consistently across different stages of the design and evaluation process.

Because the study was exploratory and context-specific, the findings should be interpreted within the examined setting and should not be considered evidence of causal effects or generalizable outcomes. Future research may extend this work by including comparison conditions, alternative communication formats, and broader participant populations.

## Figures and Tables

**Table 1:** Representative Keywords Across Identified Clusters.

Cluster	General Description	Representative Keywords
1	Design-Related Feedback	readability, text size, color contrast, animation speed, narration, layout
2	Sentiment-Related Responses	helpful, engaging, interesting, informative, emotional, useful
3	General Comments	health, depression, awareness, communication, information, discussion

**Table 2:** Mapping of design-related comments to design specifications.

Representative Comment Theme	Example User Feedback	Design Consideration	Design Specification Implemented
Readability	“The text moves too quickly to read.”	Improve readability for older adults	Larger font size and longer display duration
Visual Clarity	“Some elements are difficult to distinguish.”	Enhance visual clarity	High-contrast color combinations and simplified visual layout
Animation Pace	“The animation feels too fast.”	Reduce processing demands	Moderate animation speed and smoother transitions
Audio Support	“Narration helps explain the content.”	Reinforce information delivery	Synchronized audio narration with visual content
Content Organization	“The information is easier to follow when presented step by step.”	Improve content structure	Segmented presentation with one key message per scene

**Table 3:** Participant Characteristics.

Characteristic	Value
Number of participants	200
Mean age (SD)	51.3 (3.4) years
Age range	45–57 years
Female	108 (54.0%)
Male	92 (46.0%)
Above secondary education	100
Below secondary education	100
Regular digital media users	156 (78.0%)

**Table 4:** Cluster Selection Results.

*k*	Average Silhouette Score
2	0.39
3	0.42
4	0.36
5	0.31
6	0.28

**Table 5:** Clustering performance evaluation.

Method	Feature	Parameter	Silhouette Score	DBI
K-means	TF-IDF	*k* = 3	0.42	1.68
	Word2Vec	*k* = 3	0.38	1.84
Agglomerative	TF-IDF	*k* = 3	0.40	1.75
	Word2Vec	*k* = 3	0.36	1.92
DBSCAN	TF-IDF	eps = 0.5	0.33	2.05
	Word2Vec	eps = 0.6	0.30	2.18

Note: Higher silhouette scores indicate greater cluster cohesion and separation, whereas lower Davies–Bouldin Index (DBI) values indicate more compact and distinct clusters. In exploratory text clustering, DBI values close to 0 indicate stronger cluster separation, whereas values around 1–2 are commonly observed in datasets containing overlapping themes and vocabulary. Because user-generated comments often contain overlapping themes and vocabulary, moderate clustering performance is commonly observed in exploratory text clustering studies.

**Table 6:** Expert Validation of Extracted Design Features.

Design Feature	Mean	SD
Color contrast clarity	4.33	0.58
Animation speed	4.17	0.76
Typography readability	4.50	0.50
Audio synchronization	4.10	0.70
Content segmentation	4.27	0.64

**Table 7:** Expert Evaluation of Developed Videos.

Dimension	Mean	SD
Visual Design	4.28	0.62
Motion and Timing	4.15	0.71
Content Structure	4.32	0.55
Interaction and Usability	4.20	0.68

**Table 8:** User Evaluation Results.

Construct	Mean	SD
Cognitive Load	3.72	0.81
Usability	3.95	0.74

**Table 9:** Comparison of cognitive load and usability by educational background.

Construct	Above Secondary Education (*n* = 100) Mean ± SD	Below Secondary Education (*n* = 100) Mean ± SD	t	*p*
Cognitive Load	3.89 ± 0.40	3.85 ± 0.37	0.77	0.441
Usability	3.90 ± 0.38	3.96 ± 0.42	−1.04	0.301

## Data Availability

The data supporting the findings of this study are partially available from publicly accessible sources. The user-generated comments analyzed in this study were collected from publicly available short-form motion graphics videos on YouTube using the YouTube Data API. Due to platform policies and ethical considerations related to user privacy, the raw comment data cannot be fully shared. Processed and anonymized data, as well as additional details regarding data collection and preprocessing, are available from the corresponding author upon reasonable request.

## Appendix A. Questionnaire Items Used in User Evaluation

Participants responded to all items using a five-point Likert scale ranging from 1 (Strongly Disagree) to 5 (Strongly Agree).

A. Cognitive Load (6 Items)

CL1. The information presented in the motion graphics required considerable mental effort to understand.

CL2. I found the amount of information presented to be manageable.

CL3. The content was easy to understand.

CL4. I needed to concentrate carefully to follow the information presented.

CL5. The presentation format helped me to process the information.

CL6. I felt comfortable following the information throughout the video.

B. Usability (6 Items)

US1. The text and visual elements were clear and easy to read.

US2. The organization of the content was easy to follow.

US3. The animation speed was appropriate for understanding the information.

US4. The audio narration supported my understanding of the content.

US5. The motion graphics were easy to interact with and follow.

US6. Overall, I was satisfied with the presentation of the information.
